# Arthroscopically-assisted reduction and pinning of a radial neck fracture in a child: a case report and review of the literature

**DOI:** 10.1186/s13256-020-02390-0

**Published:** 2020-06-25

**Authors:** Alessandra Colozza, Sara Padovani, Gaetano Caruso, Michele Cavaciocchi, Leo Massari

**Affiliations:** 1 Orthopaedic and Traumatology Unit, Azienda Ospedaliera di Faenza, Viale Stradone 9, 48018 Faenza, RA Italy; 2 Department of Orthopaedic and Traumatology Surgery, Azienda Ospedaliero-Universitaria S. Anna Cona, University of Ferrara, Via Aldo Moro 8, 44124 Ferrara, Italy

**Keywords:** Radial neck, Elbow, Arthroscopy, Fractures, Children

## Abstract

**Background:**

Displaced radial neck fractures in children are challenging to treat. The age of the patient and the degree of angulation are the main criteria to consider when managing these fractures in children. Various surgical techniques have been described in the literature for both indirect and direct reduction and for fixation. However, the best treatment is still debated.

**Case presentation:**

The case presented is of a 6-year-old Caucasian boy with an impacted and displaced radial neck fracture. With the patient in lateral position, under general anesthesia, elbow arthroscopy was performed to better visualize the articular surface and to assist with reduction and fixation. The fracture was reduced and fixed with a single K-wire under direct arthroscopic visualization. No associated lesions were found. An above-elbow cast was applied after surgery. The cast and K-wire were removed 3 weeks later. At the 3-month follow-up, the patient showed a full recovery with complete range of movement without any postoperative and radiographic complications.

**Conclusion:**

Traditionally, surgery for displaced radial neck fractures in children is performed by closed reduction with percutaneous pinning or elastic intramedullary nail fixation under fluoroscopic guidance. Direct visualization of the articular surface via an open approach allows better reduction in complex fracture patterns but is related to a higher risk of complications: elbow stiffness, instability, or avascular necrosis. Elbow arthroscopy in children could be a valid alternative to open fixation surgery for displaced radial neck fractures without the complications associated with articular exposure, allowing the direct visualization of the fracture and reducing radiation exposure. Although technically demanding, we believe elbow arthroscopy should be considered an alternative option because it is effective in assisting reduction and fixation and enables the detection of associated joint lesions.

## Background

Fractures of the neck of the radius account for 5% of elbow injuries and 1% of all injuries in children [1]. These fractures frequently affect children between the ages of 4 and 14, because radial head ossification starts at age 5 and is complete when the physis closes at 14 years of age for females and at 17 for males. There are two anatomic types of radial neck fracture. The most common is by far the simple metaphyseal fracture of the radial neck with subsequent Salter type II epiphyseal separation. The other type of epiphyseal separation is rare and characterized by intra-articular fracture extension (Salter types III and IV) [2]. Many classification systems have been developed for radial neck fractures: The Jeffery, O’Brien, and Judet and Letournel classification systems are the most commonly cited ones in the literature. According to Jeffery, group I is the most common injury type, resulting from impaction of the capitellum against the radial head during a fall on an outstretched open hand, where the elbow is extended or slightly flexed and a valgus force is applied to the elbow joint. Group II fractures occur as a result of an elbow dislocation or subluxation with an angulation of the radial neck of up to 90 degrees in the direction of dislocation. In this case, the radial head remains in an anterior position [3]. This form of fracture adversely affects the prognosis because of the associated vascular risk. In the O’Brien classification, type I fracture lines of the proximal and distal fragments have an angulation of less than 30 degrees, type II fractures range from an angulation of 30 degrees to 60 degrees, and type III fractures have an angulation that exceeds 60 degrees. The Judet and Letournel classification is perhaps the most widely used. Grade I fractures involve translation of the proximal radial epiphysis with no angulation; grade II fractures have an angulation < 30 degrees, grade III fractures range from 30 degrees to 60 degrees; grade IV fractures range from 60 degrees to 80 degrees; and grade V fractures exceed 80 degrees [4]. During displacement, a metaphyseal periosteal flap often remains attached to the radial head. The preservation of this sleeve, which contributes to the vascular supply to the radial head, is critically important for two reasons: first, because it preserves the blood vessels it contains; second, because, once tensioned, this piece of tissue will assist in maintaining the reduction. The treatment of radial neck fractures in children varies according to the fracture displacement (lateral shift), the angulation, and skeletal maturity. It is generally agreed that radial neck fractures with < 3-mm displacement and < 30-degree angulation (Judet and Letournel type I or II) should be treated conservatively or with closed reduction by manipulation and cast immobilization, whereas for fractures with higher degrees of angulation and displacement (Judet and Letournel types III and IV), surgery is recommended [5]. Various surgical techniques have been described in the literature for reduction and for fixation. These include the Kirschner wire or Steinman pins, which are used to manipulate the radial head into place under fluoroscopy or, rarely, arthroscopy, the Métaizeau technique, or, in case of a complex fracture or secondary displacement, open reduction, considered to be the last step in the management cascade but associated with worse outcomes. The prognosis of these fractures is related to several factors, such as the angulation of the radial head, the mechanism of injury, the type of epiphyseal plate disruption, the amount of fracture displacement, the open surgical approach, or a combination of these. Reported complications of these injuries in children include elbow stiffness (which occurs in 10–31% of cases) [6], avascular necrosis (which may occur in up to 10% of all patients and up to 25% of patients requiring an open reduction) [7], and synostosis and overgrowth of the head of the radius, which lead to persistent lateral elbow pain and restricted elbow range of motion [1].

To our knowledge, only two authors [8, 9] have described elbow arthroscopy as a useful and alternative technique to achieve good reduction of the proximal radial epiphysis. The aim of the present case report is to describe an impacted and minimally displaced radial neck physis fracture fixed by K-wire under elbow arthroscopy in a young boy.

## Case presentation

**Fig. 1:**
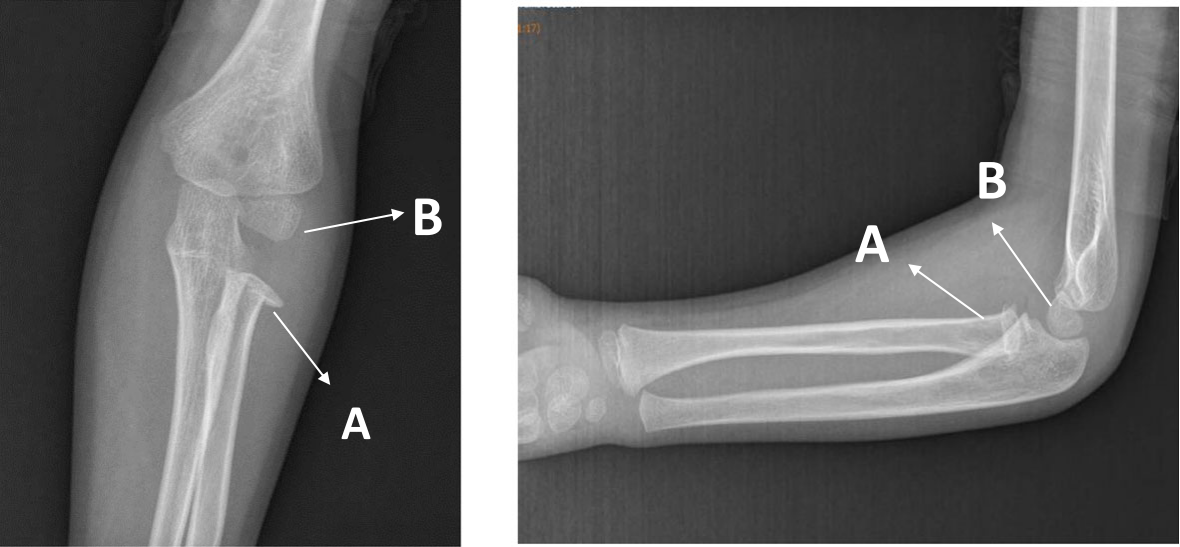
Pre-operative anteroposterior (AP) and lateral x-ray view of the left elbow. A: the radial neck, displaced laterally; B: the capitulum humeri. Notice the abnormal gap between A and B in the AP view

**Fig. 2:**
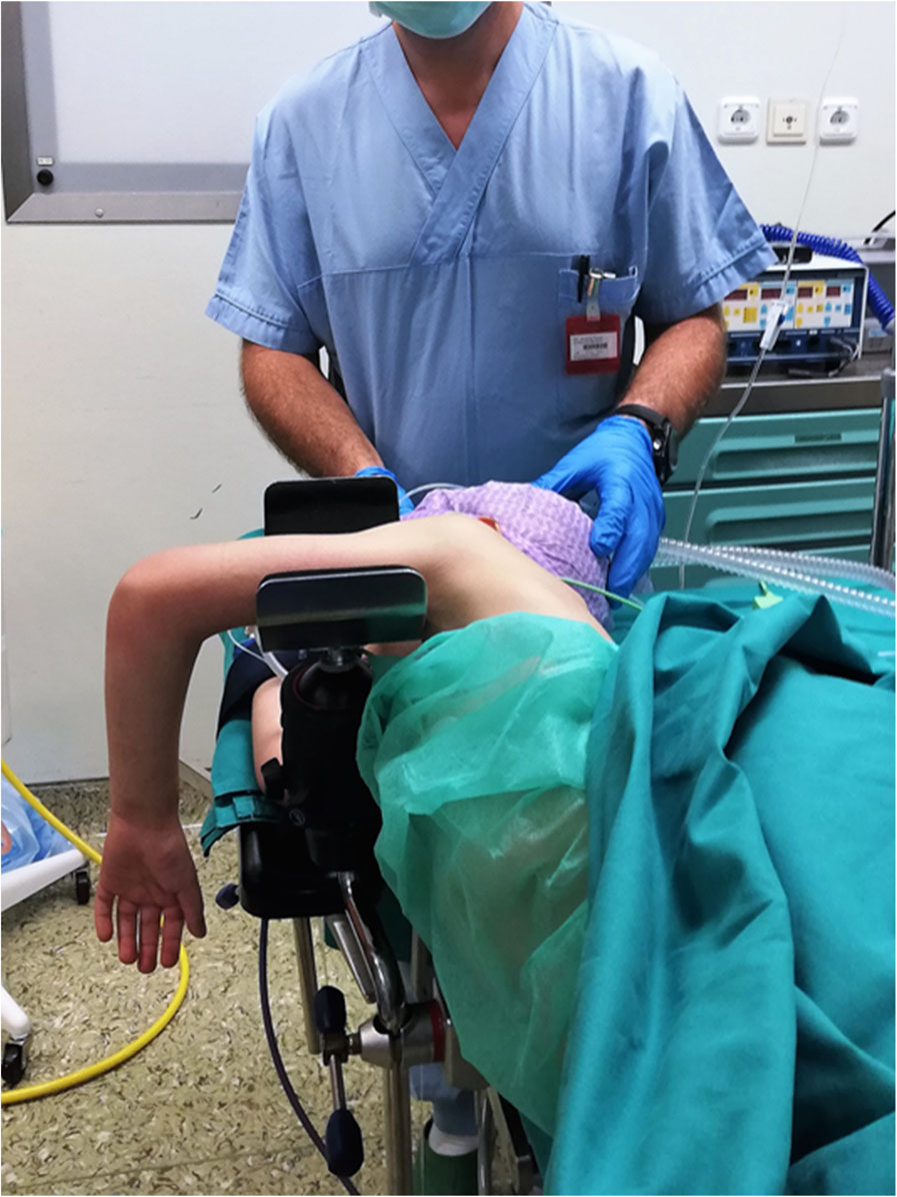
Patient under general anaesthesia in lateral decubitus with the elbow at 90° of flexion

**Fig. 3:**
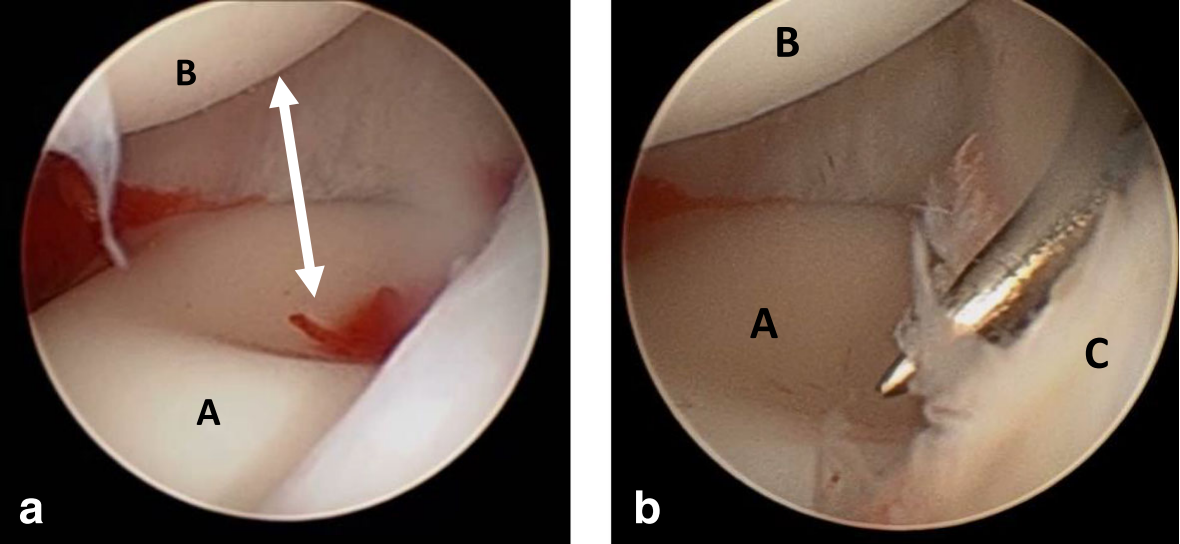
(**a**, **b**) Arthroscopic images before fracture reduction. Notice the abnormal distance (white arrow) between the radial head (A) and the capitulum humeri (B), which should normally be in contact. The condropick arthroscopic instrument easily passes through the articular space over the annular ligament (C) between the capitulum humeri and the radial head; this is an indirect sign of articular incongruity

**Fig. 4:**
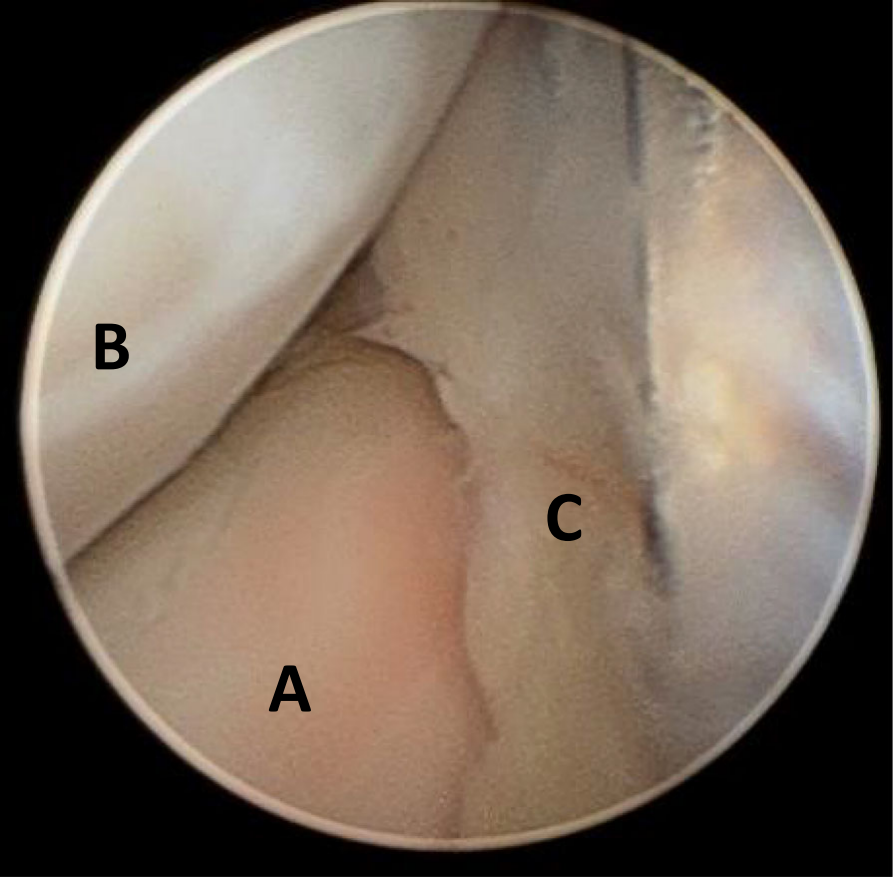
Arthroscopic image, after reduction and fixation, showing the normal relation-contact between the proximal radio-humeral joint. Radial head (A) capitulum humeri (B) and intact annular ligament (C)

**Fig. 5:**
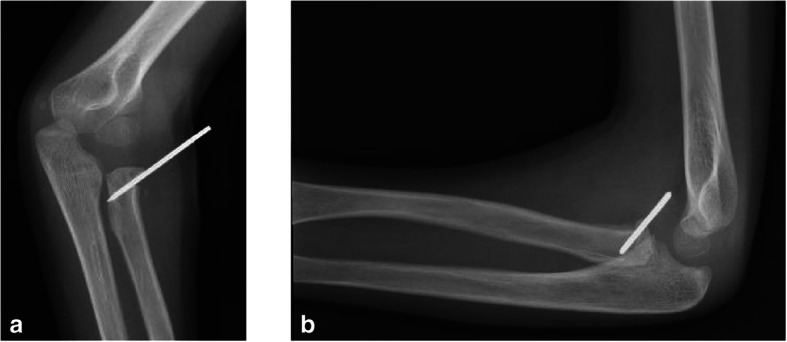
Oblique (**a**) and lateral (**b**) x-ray of the left elbow after reduction and fixation

The patient’s family provided written informed consent to publish this case report and any accompanying images. A 6-year-old Caucasian boy fell off his bike in the spring and landed on his outstretched arm, producing a valgus thrust on the elbow that fractured the radial neck. The patient arrived at the emergency department the same day of the injury, reporting acute left elbow pain and inability to move it. The elbow was swollen and painful laterally, with no clinically evident deformities of the left upper arm and only a small abrasion on his left hand. There was no neurovascular deficit of the upper limb and no sign of elbow instability. Radiographs showing anteroposterior and lateral projections of the left elbow revealed a radial neck metaepiphyseal impacted fracture with a displacement of 4 mm and an angulation < 30%, classifiable as a Judet and Letournel type II fracture (Fig. 1). The patient’s relatives accepted the treatment proposed after having been informed that the fracture, impacted and displaced, often results in a loss of forearm rotation if not treated. The patient was hospitalized for a routine preoperative checkup. His blood examination and anesthesia evaluation were carried out. The limb was placed in a splint at 90 degrees of flexion, and surgery was performed the following day. Once general anesthesia was performed, the manipulation of the left elbow was attempted with the elbow being flexed and the forearm supinated. Posteriorly directed pressure was applied to the radial shaft as described by Monson *et al.* [10]. A fluoroscopy check showed that the displacement had not healed and that the fracture was still impacted. The operating room setup was similar to the one used for elective elbow arthroscopy in adults. The patient was placed on the operating table in the lateral decubitus position, using a tourniquet and maintaining the elbow at 90-degree flexion during surgery (Fig. 2). A fluid pump was used at low pressure. Bony landmarks (radial head, epicondyles, and olecranon process) were marked, and the joint was distended with an intra-articular injection of 5 ml of saline solution through the posterior portal (the olecranon fossa). The anteromedial portal was used as the arthroscope entrance, and the anterolateral portal was used as the instrumental portal. Intra-articular evaluation of the joint showed the integrity of the annular ligament and a significant displacement and impaction of the radial head detectable as an increased gap between the radiohumeral joint, which was enhanced by pronation and supination movements (Fig. 3). The proximal anteromedial portal was made 2 cm proximal and 1 cm anterior to the medial epicondyle to avoid injury to the ulnar nerve. The skin was carefully incised, and the soft tissues were bluntly dissected down to the level of the capsule. A 2.4-mm 30-degree arthroscope was inserted through this portal, and another portal was made under direct visualization with an outside-in technique. Using an anterolateral arthroscopic portal, a Kelly hemostatic forceps was introduced and used to gain the reduction of the radial head under arthroscopic assistance, but without success. Some force had to be applied to perform the reduction using a K-wire inserted percutaneously as a lever (joystick technique). The result was graded as good (Fig. 4). A dynamic arthroscopic examination showed satisfactory stability of the osteosynthesis and normal articular congruity. A single check with an image intensifier, in anteroposterior and lateral views, was taken at the end of the procedure (Fig. 5). The surgery lasted 35 minutes. Postoperatively, a cast was applied for 3 weeks, and then the K-wire was removed. The postoperative course was uneventful, and the fracture was united at 4 weeks. Three months after the fracture event, the patient had regained full range of motion with a Mayo Elbow Performance Score of 100 points.

## Discussion

Radial neck fractures in children fall under a spectrum of injuries that should be addressed using an algorithm of progressive intervention: from closed manipulation to percutaneous fixation to open attempts, with the aim being to gain the best acceptable reduction [11]. The present report describes a case of an impacted, displaced, and minimally angulated radial neck fracture in a 6-year-old child, treated by reduction and fixation with K-wire under-elbow arthroscopy after an unsuccessful manipulation attempt. Restoration of radial neck angulation and alignment is essential to reestablish the normal biomechanics and stability of the elbow. The standard for minimally angulated (< 30-degree) fractures in young patients is simple immobilization, which often results in excellent outcomes for most patients. However, many classifications do not consider the impaction of the fracture and the displacement in the coronal plane of the proximal radial epiphysis. Indications for the treatment of radial neck fractures should consider several factors: fracture location, degree of displacement, associated injuries, skeletal maturity, and time elapsed since the injury. The amount of angulation is still considered the primary criterion in the decision for a conservative or surgical treatment of fracture management. Kim *et al.* reported that an angulation < 30 degrees can be treated by simple immobilization and casting, an angulation of 30–60 degrees by closed reduction and fixation or by open reduction if the closed reduction fails, and an angulation ≥ 60 degrees by open reduction [9]. The age of the patient and the degree of skeletal maturity are also critical when defining the treatment indication; for example, older children with radial neck fractures show worse outcomes than younger children. Métaizeau *et al.* reported that an angulation of 20–30 degrees in young children may be remodeled in time, but even an angulation of 10–15 degrees in children over 12 years old cannot be remodeled [12]. Similarly, Bernstein *et al*. reported that remodeling is possible in angulations of 60 degrees in children up to 6 years of age, but angulations > 30 degrees cannot be corrected in children older than 12 years [13]. However, the management of pediatric radial neck fractures, especially of those with an angulation of > 30 degrees or a displacement > 3 mm, is still controversial. For this reason, the treatment of choice for pediatric radial neck fractures is yet to be established [9]. These fractures can be challenging to reduce, and this is the reason why many surgical options have been described, including indirect reduction and fixation techniques under fluoroscopic assistance, such as percutaneous pinning and the elastic stable intramedullary nailing (ESIN) technique or direct technique by open reduction with or without internal fixation and suturing of the annular ligament, more common in the past but still used for unsuccessful indirect reduction. The ESIN technique, better known as the Métaizeau technique, is a minimally invasive surgery performed with a single nail contoured and bent at the tip, approximately 30–45 degrees, to catch the displaced proximal epiphysis, whereas the rest of the nail is prebent with a gentle curve of 20 degrees to obtain a three-point fixation. Under image intensifier guidance, the nail is introduced proximally to the distal radial epiphysis, advanced proximally with gentle rotational movements until it reaches the fracture, and then rotated until the fracture is reduced [12]. The percutaneous pinning technique consists of a K-wire inserted percutaneously from the proximal to distal radial neck fracture site, which is used as a lever, and the radial neck fracture is reduced under image intensifier guidance [14]. After reduction, the K-wire is advanced toward the ulnar side to impact the opposite cortex. The stability of reduction and forearm rotation is checked under fluoroscopic control. In two studies comparing the Métaizeau technique and percutaneous pinning [15, 16], the authors found no statistical superiority of one technique over the other in terms of clinical outcome and radiological alignment. Direct visualization of the joint surface requires open surgery to obtain the best reduction, but it is widely reported that open reduction by articular exposure leads to worse medium-term and long-term functional outcomes [16]. The open surgical approach is achieved by using the Kocher approach between the anconeus and the extensor carpi ulnaris; the radial head is gently repositioned and the interposed capsular or ligamentous structures that blocked reduction are removed. If the head fragment is unstable, fixation using Kirschner wire or Steinmann pins is necessary.

Higher rates of complications are reported after open surgery than after closed reduction, especially with regard to the incidence of avascular necrosis (19% vs. 5%), premature epiphyseal fusion (50% vs. 5%), and heterotopic ossification (25% vs. 4%) [15], although it is unknown how much of the poor outcome is attributable to the open reduction rather than to the worse nature of the injury. In a large series of surgically treated radial neck fractures, Basmajian *et al.* reported a 35% rate of good or excellent outcomes with open reduction compared with a 73% rate of good or excellent outcomes with percutaneous reduction [17]. In a series of 24 fractures treated with open reduction after failed closed reduction, Falciglia *et al*. reported 25% fair and 20% poor outcomes associated with a loss of forearm rotation, loss of flexion-extension, increased valgus carrying angle, osteonecrosis of the radial head, and premature physeal closure [18]. Direct surface radiocapitellar joint visualization could also be achieved by elbow arthroscopy as an alternative to open reduction or in case of unsuccessful manipulation. On the one hand, elbow arthroscopy is known as a safe and effective surgical procedure in the adult population, with surgical indications increasing in recent years; on the other hand, there are technical skills required to avoid proximal major neurovascular structure and limit joint capsule range. In children, this approach is even more challenging due to the anatomy and size of the elbow joints [19]. The lateral and medial epicondyles used as bony landmarks in the general elbow scope can be tricky to detect in children because the formation of the ossification center varies with age. To our knowledge, there are only two studies in the literature reporting radial neck fractures treated by elbow arthroscopy [8, 9]. The first one is a case report published in 2004, in which an 11-year-old girl underwent elbow arthroscopy and percutaneous pinning for a type III radial neck fracture with good results at the 2-year follow-up [8]. The other one is a case series of seven patients treated by arthroscopically assisted percutaneous pinning after unsatisfactory reduction due to failed closed reduction. The authors reported the radial neck angulation preoperative (mean 48.8 degrees) and postoperative values (mean 1.7 degrees), with good to excellent outcomes assessed according to the Mayo Elbow Performance Score [9]. Dawson and Inostroza and Kim *et al*. achieved good results by treating radial neck fractures in children under arthroscopic guidance, finding this procedure safer and having a lower risk of postoperative complications than open approaches [8, 9]. Moreover, the reduction and fixation guided by arthroscopy allow direct visualization of the articular surface, better evaluation of associated elbow lesions, and a chance to treat them, which is not possible with the traditional indirect techniques outlined above. Dawson and Inostroza [8] and Kim *et al*. [9] performed arthroscopy with their patients in prone position, without using a tourniquet and fluid pump, considering the younger age of the population; in our patient’s case, however, the experienced arthroscopist who performed the procedure used the lateral decubitus position with the elbow at a 90-degree angle and a tourniquet in order to obtain the best visualization possible.

At the final clinical and radiographic follow-up, the patient had no complications, such as heterotopic ossification, avascular necrosis, or premature epiphyseal fusion described in the literature, and he had gained a complete range of movement without the need for physical therapy.

Finally, arthroscopic treatment should not be considered a time-consuming technique. The surgery performed on our patient lasted 35 minutes, whereas traditional surgical operations are estimated to last 25.6 minutes in closed reduction by percutaneous pinning and 51 minutes with the Métaizeau technique. Moreover, in our patient’s case, the radiation exposure dosage was drastically reduced to as little as 5 seconds, compared with durations reported for percutaneous pinning and ESIN of 18 and 53 seconds, respectively [16].

## Conclusion

Elbow arthroscopy in children may be considered an alternative technique to assist reduction and fixation of displaced and moderately angulated radial neck fractures or in case of unsuccessful indirect reduction. Arthroscopy, although technically demanding, has several advantages over traditional surgery: direct visualization of the entire joint surface and better evaluation of associated lesions, thus reducing radiation exposure for young patients and operative staff.

## Data Availability

The dataset supporting the conclusions of this report is available in the archive of the Orthopaedic and Traumatology Unit, Azienda Ospedaliero-Universitaria di Ferrara Arcispedale Sant’ Anna, University of Ferrara, Via Aldo Moro 8, 44124 Ferrara, Italy.

## Abbreviation

ESIN: Elastic stable intramedullary nailing
